# Hormonal contraception in women with migraine: is progestogen-only contraception a better choice?

**DOI:** 10.1186/1129-2377-14-66

**Published:** 2013-08-01

**Authors:** Rossella E Nappi, Gabriele S Merki-Feld, Erica Terreno, Alice Pellegrinelli, Michele Viana

**Affiliations:** 1 Research Center for Reproductive Medicine, Gynecological Endocrinology and Menopause, IRCCS S. Matteo Foundation, Pavia, Italy; 2 Department of Clinical, Surgical, Diagnostic and Paediatric Sciences, University of Pavia, Pavia, Italy; 3 Clinic for Reproductive Endocrinology, University Hospital Zürich, Zürich, Switzerland; 4 Headache Science Center - National Neurological Institute C. Mondino, Pavia, Italy; 5 Research Center for Reproductive Medicine, Unit of Obstetrics and Gynecology, IRCCS Policlinico ‘San Matteo’, Piazzale Golgi 2, 27100, Pavia, Italy

**Keywords:** Migraine with aura (MA), Migraine without aura (MO), Combined hormonal contraceptives (CHCs), Combined oral contraceptives (COCs), Progestogen-only contraception, Desogestrel-only pill, Venous thromboembolism (VTE), Stroke

## Abstract

A significant number of women with migraine has to face the choice of reliable hormonal contraception during their fertile life. Combined hormonal contraceptives (CHCs) may be used in the majority of women with headache and migraine. However, they carry a small, but significant vascular risk, especially in migraine with aura (MA) and, eventually in migraine without aura (MO) with additional risk factors for stroke (smoking, hypertension, diabetes, hyperlipidemia and thrombophilia, age over 35 years). Guidelines recommend progestogen-only contraception as an alternative safer option because it does not seem to be associated with an increased risk of venous thromboembolism (VTE) and ischemic stroke.

Potentially, the maintenance of stable estrogen level by the administration of progestins in ovulation inhibiting dosages may have a positive influence of nociceptive threshold in women with migraine. Preliminary evidences based on headache diaries in migraineurs suggest that the progestin-only pill containing desogestrel 75 μg has a positive effect on the course of both MA and MO in the majority of women, reducing the number of days with migraine, the number of analgesics and the intensity of associated symptoms. Further prospective trials have to be performed to confirm that progestogen-only contraception may be a better option for the management of both migraine and birth control. Differences between MA and MO should also be taken into account in further studies.

## Introduction

Migraine is a disabling headache, characterized by moderate to severe head pain, usually accompanied by nausea, photophobia, phonophobia and osmophobia (migraine without aura, MO). In about 30% of patients migraine attacks are preceded by transient focal neurologic symptoms which are called aura (migraine with aura, MA). Migraine has a high socio-economical impact. In fact during migraine attacks most migraineurs reported severe impairment or the need of the bed rest and almost 40% of migraine patients have five or more headache days monthly [1]. The Global Burden of Disease survey 2010 (GBD) recently published showed that migraine is the seventh highest cause of disability in the world [2].

## Review

In the last few years significant advance in the field of reversible hormonal methods has been achieved in order to maximize the benefits and to minimize the risks.

We believe it is relevant for clinical practice to briefly review in here potential vascular risks according to the category of migraine, with and without aura, and to the type of hormonal contraceptive option.

### Epidemiology of migraine and combined hormonal contraceptive (CHC) use

Migraine affects about 18% of women and 6% of men in USA and Western Europe [3,4] and its cumulative lifetime prevalence is 43% in women and 18% in men [5]. It is then mostly a female disorder, that it is active in particular during the fertile period of the women’ life with a peak of prevalence in their 20s and 30s [6]. The reproductive life is characterized by the need of reliable and convenient methods of contraception. Among the several forms of contraceptives available, hormonal contraceptives are the most popular reversible method, both in USA and Europe and the “Pill” is the most used [7-9]. Low dose [20 to 30 μg ethynil estradiol (EE2) per day] combined hormonal contraceptives (CHCs) have become the method of choice and the availability of new progestins (third- and fourth-generation) has allowed to achieve non-contraceptive benefits in comparison to older progestins (second – generation) [10]. CHCs are available in several regimens and routes of administration (oral, transdermal vaginal) in the attempt to improve tolerability, adherence and convenience of use [11]. Moreover, two new CHCs containing natural estradiol (E2), instead of EE2, have been introduced to increase safety and future developments are ongoing [12]. Interestingly, many gynecological conditions that are comorbid with migraine can be treated with CHCs. This enhances the likelihood of their use in migraine population [13]. The prescription of CHCs may have different effects on migraine with not univocal results because of many methodological limitations (diverse hormonal combinations, variable research settings, retrospective and/or cross-sectional designs, lack of a clear phenotyping of the headache according to IHS criteria, inadequate duration of observation) [14-16]. Historically, combined oral contraceptive (COCs) is the category best studied in migraineurs with an aggravation of migraine reported in 18-50% of cases, an improvement in 3-35% and no change in 39-65% [17]. A more recent cross-sectional study on a large population found that migraine is significantly associated with COCs assumption. Yet because of the design of the study it is not possible to define a causal relationship between exposure and disease [18]. Analysis on the different effect of the COCs on the two forms of migraine revealed that MA worsen more (56.4%) than MO (25.3%) [19]. Furthermore, women can present MA for the first time during the initiation of COCs [20]. During the last decade, a specific “window” of vulnerability triggered by the 7 days free hormone interval has been identified and the definition of hormonally-associated headaches (exogenous hormone-induced headache and estrogen-withdrawal headache) encompasses several patho-physiological mechanisms which are likely to explain nociceptive threshold in women [21,22]. Strategies to minimize estrogen withdrawal at the time of expected bleeding or to stabilize circulating estrogen at lower concentration include, respectively, 1) to use transdermal E2 during the free interval of hormonal contraception [22] or to shorten the interval from 7 to 4 and even to 2 days [23,24]; 2) to administer low dose COCs in extended/flexible regimens [25] or to use extended vaginal contraception [26].

Very recently, Mac Gregor [27] reviewed the effects of currently available contraceptive methods in the context of the risks and benefits for women with migraine and non-migraine headaches and concluded that for the majority of women with headache and migraine, the choice of contraception is unrestricted. Indeed, the contraceptive method is unlikely to have an impact on headache, whereas migraine deserves accurate diagnosis and recognition of the impact of different methods on such condition.

### Vascular risks associated with CHCs and migraine

MA, and to a lesser extent also MO, may increase vascular risk, especially the risk for ischemic stroke in younger women [27-30]. Moreover, evidences that need to be corroborated by further studies suggest an association between MA and cardiac events, intracerebral hemorrhage, retinal vasculopathy and mortality [31]. Even though the association between migraine and stroke appears to be independent of other cardiovascular risk factors [32], the presence of some risk factors, such as smoking and/or COCs use or their combination, further increase risk [33]. MA is associated with a twofold increased risk of ischemic stroke but the absolute risk associated with CHC use is very low in healthy young women with no additional risk factors and mostly related to the estrogen dose [34]. In spite of the considerable advances in terms of safety and tolerability of CHCs in migraine sufferers [13], their use is still questioned especially in women with additional risk factors for stroke, including, smoking, hypertension, diabetes, hyperlipidemia and thrombophilia, age over 35 years [35]. New evidences [36-38] have warned clinicians on the use of CHCs and the risk of venous thromboembolism (VTE) which is likely to be dependent on the type of the progestin [RR 1.6–2.4 by using third- and fourth-generation CHCs (namely, desogestrel, gestodene norgestimate and drospirenone, respectively) in comparison with those containing LNG (second-generation)] and the total estrogenicity of CHCs [39,40]. Even new routes (trandermal patch and vaginal ring) seem to be associated with an increased VTE risk, but data are contradictory [41-43]. Indeed, according to a statement very recently released [44] many factors contribute to VTE risk (e.g. age, duration of use, weight, family history) which makes epidemiological studies vulnerable to bias and confounders. In addition, the decision-making process should take into account non only the small VTE risk (absolute risk depending on the background prevalence rate between 2 to 8 per 10,000 users per year [45]) of the contraceptive method but also other elements such as efficacy, tolerability, additional health benefits, and acceptability which have to be discussed with the individual woman. In any case, it is essential to follow the appropriate guidelines by avoiding the prescription of CHCs to women at elevated risk for VTE. In the context of migraine and CHCs use, it is very important to remember that the World Health Organization Medical Eligibility Criteria for Contraceptive Use stated that MA at any age is an absolute contraindication to the use of COCs (WHO Category 4) [46]. The US/WHO MEC is more restrictive than the UK/WHO MEC as regard to MO, rating CHCs as a category 4 for any migraineur over age 35 [47]. That being so, the personal risk assessment should guide the prescription of CHCs in selected conditions. When there is the sole need of contraception, without the added benefits of a peculiar hormonal compound and/or combination, CHCs with the lower vascular risk or alternative methods for birth control should be considered.

### Progestogen-only contraception: a class on its own

The progestin component of hormonal contraceptives accounts for most of their contraceptive effects (inhibition of ovulation, suppression of endometrial activity, thickening of cervical mucus). Progestin-only methods includes pills (the pill most used in Europe contains low doses of desogestrel), injectables [depot Medroxyprogesteroneacetate (DMPA)], implants (the most recent long-acting reversible contraception contains Etonogestrel single-rod implant for at least 3 years), and intrauterine devices (levonorgestrel for at least 5 years) [48]. By providing effective and reversible contraception, progestin-only contraception has many noncontraceptive health benefits including improvement in dysmenorrhea, menorrhagia, premenstrual syndrome, and anemia [49]. Indeed, there is a general reduction of the amount of menstrual bleeding but cycle control may be erratic, a feature that may influence acceptability [50-52]. Progestin-only methods are appropriate for women who cannot or should not take CHCs because they have some contraindications to estrogen use and therefore display a higher risk of VTE [35,36]. The progestogen-only contraception is a safe alternative to CHCs and the avoidance of the estrogen component has many advantages not only for breastfeeding women but also for women with vascular diseases or risk factors for stroke [46,47]. The use of progestin-only contraception is not associated with an increased risk of VTE compared with non-users of hormonal contraception [53]. In addition, progestin-only pills, injectables, or implants are not associated with increased risk of ischemic stroke according to a recent metanalysis (OR 0.96; 95% CI: 0.70-1.31) [54]. Since the 1-year prevalence rates for migraine in women are 11% for MO and 5% for MA, respectively [55], there is potentially a high number of women in whom CHCs may be contraindicated according to WHO guidelines and progestogen-only contraception may be safely used [35,36].

### Evidence of progestogen-only contraception in women with migraine

Given the evidence that progestogen-only contraception is a safer option for women with migraine, the main question is whether such contraceptive choice may influence the course of both MA and MO and offer a better management of the disease. Indeed, even though the excess risk of death for a woman taking modern CHCs is 1 in 100,000, which is much lower than the risk of everyday activities such as cycling [56], there is a biological plausibility that in women with migraine should be wiser to use an estrogen-free containing contraception to avoid any potential vascular risk. Two recent very large epidemiologic studies [36,57] reported the association between CHC and progestogen-only methods and cardiovascular risk, thrombo-embolic risk and stroke. Whereas no increased risk for deep venous thormbosis, myocardial infarction and thrombotic stroke was found for the progestogen-only methods, the risk were two-sixfold elevated in CHC users. The role of progesterone/progestins in the pathophysiology of migraine has been overshadowed by Somerville’s early observations that it was the prevention of estrogen but not of progesterone withdrawal in the late phase of the cycle to be able to prevent the occurrence of migraine attacks [58,59]. Indeed, at variance with the influence of estrogens upon the cerebral structures implicated in the pathophysiology of migraine [60], cyclic variations in progestin levels were not related to migrainous headaches, but they rather seem to be protective. Progesterone apparently attenuates trigemino-vascular nociception [61] and its receptors are localized in areas of the central nervous system, which are involved in neuronal excitability and neurotransmitter synthesis release and transport [62]. It has been shown that progesterone can antagonize neuronal estrogenic effects by downregulating estrogen receptors [63]. Whereas estrogen peak decrease the threshold for cortical spreading depression (CSD), the neurobiological event underlying MA, estrogen withdrawal increased the susceptibility to CSD in an animal model [64]. Therefore, the maintenance of low estrogen levels and the avoidance of estrogen withdrawal by the administration of progestins in ovulation inhibiting dosages might decrease cortical excitability. Indeed, progestogen-only contraception has a continuous administration, without the hormone-free interval, and does not induce withdrawal stabilizing circulating estrogens, but some fluctuations according to different preparations may still occur [65]. Clinical data are scarce and no comparative studies with progestogen-only contraceptives and placebo or COCs are available in the literature [27]. Diagnoses are often inaccurate, without distinction between headache and migraine, and headache is reported in contraceptive progestin implant users as a potential cause of discontinuation [66]. Similarly, there is an increase in headache, but not migraine, reported over time with both norethisterone enanthate and, especially with depot medroxyprogesterone acetate [67]. Anecdotally, migraine is more likely to improve in women who achieve amenorrhea [68]. In a large, cross-sectional, population-based study in Norway of 13944 women, a significant association between CHC and headaches, but no significant association between progestin-only pills and migraine (OR 1.3, 95% CI: 0.9–1.8) was found but the number of users was small [18]. To date two diary-based studies pilot studies on the effect of desogestrel 75 μg on migraine have been published [69,70]. Such oral daily pill inhibits ovulation and the dose allows the ovary to synthesize stable amounts of estrogen which are relevant for wellbeing and bone density [71]. The first study included thirty women with MA [69]. The use of desogestrel 75 μg resulted in a significant reduction in MA attacks and in the duration of aura symptoms, already after three months of observation. Interestingly, the beneficial effect of desogestrel 75 μg on visual and other neurological symptoms of aura was significantly present only in those women in whom MA onset was related to previous COCs treatment. These findings suggest that the reduction in estrogen levels may be relevant to the amelioration of MA, but do not exclude a direct effect of the progestin on CSD. The second study on the effect of desogestrel 75 μg included women with MA (n°=6) and with MO (n°=32) and evaluated migraine days, pain score and pain medication [70]. An improvement of each parameter was observed during 3 months use of desogestrel 75 μg in comparison to a three months pretreatment interval. A subanalyses of the effect on 32 women with MO revealed significant improvements in number of migraine days, pain medication and pain intensity. The mean number of migraine attacks at baseline was higher in comparison to that in the study of Nappi et al. [69], indicating that also very severe migraineurs might profit from such a progestin-only contraception. Chronic migraineurs often develop medication overuse headaches with severe limitation of their quality of life. The reduction of pain medication by progestin-only contraception is an interesting approach and it should be studied further. Indeed, there is a broad variation in the intensity of improvement with a reduction in migraine frequency ranging from 20% and 100% [70]. No indicators to identify those women who will profit from desogestrel 75 μg could be ascertained. On the other hand, there were few dropouts of women experiencing more migraine after starting contraception with this progestin in both studies, indicating that progestins can also deteriorate migraine in few cases.

A very recent study investigating the changes of quality of life in migraineurs 3 months after initiation of the progestagen-only pill desogestrel 75 μg demonstrates a highly significant reduction in Midas Score and Midas grades [72]. However, clinical experience with desogestrel 75 μg in migraineurs further shows that during the initial 4 weeks migraine frequency can raise slightly before headaches improve. This information has to be mentioned and discussed during counseling.

In summary, the potential advantages of using progestogen-only contraception in women with migraine are the following:

1) Continuous use

2) Absence of estrogen peak

3) No influence on threshold for cortical spreading depression (CDS)

4) No evidence of increase in cardiovascular, stroke and thrombo-embolic risk

5) No data on progestins inducing migraine

## Conclusions

In conclusion, contraceptive counseling in migraine should take into account the risk-benefit profile of the individual woman before prescribing CHCs. In order to reduce potential vascular risks, recommendations should be a main point of guidance for prescribers. Progestogen-only contraception is a safer option in MA and eventually in MO with additional risk factors. Given preliminary evidences of a positive effect of the progestin-only pill desogestrel 75 μg on MA and MO in many, but not all women, further prospective trials have to be performed to confirm that progestogen-only contraception may be a better option for the management of both migraine and birth control. Differences between MA and MO should also be taken into account in further studies.

## Competing interests

Dr. Rossella E. Nappi has had financial relationships (lecturer, member of advisory boards, and/or consultant) with Bayer Pharma, Eli Lilly, Gedeon Richter, HRA Pharma, MSD, Novo Nordisk, Pfizer Inc., Shionogi Limited, Teva/Theramex.

Dr. Gabriele S. Merki-Feld has had financial relationships (lecturer, member of advisory boards) with Bayer Pharma and MSD.

## Authors’ contributions

Conception and design: REN, GSM. Acquisition of data: ET, AP. Analysis and interpretation of data: REN, MV, GSM. Drafting the article: REN, MV, GSM. Revising it for intellectual content: REN, MV, GSM. Final approval of the completed article: REN, REN, GSM, ET, AP, MV. All authors read and approved the final manuscript.
